# Identification and Characterization of ABCG15—A Gene Required for Exocarp Color Differentiation in Pear

**DOI:** 10.3390/genes14091827

**Published:** 2023-09-21

**Authors:** Simeng Zhang, Jiayu Xu, Ying Zhang, Yufen Cao

**Affiliations:** Research Institute of Pomology, Chinese Academy of Agricultural Sciences, Xinghai South Street 98, Xingcheng 125100, China; zhangsimeng2015@163.com (S.Z.); aa881031@sina.com (J.X.); wodeying1314@163.com (Y.Z.)

**Keywords:** genome-wide association study (GWAS), transcriptome, metabolome, cuticle, suberin

## Abstract

Exocarp color is a commercially essential quality for pear which can be divided into two types: green and russet. The occurrence of russet color is associated with deficiencies and defects in the cuticular and epidermal layers, which affect the structure of the cell wall and the deposition of suberin. Until now, the genetic basics triggering this trait have not been well understood, and limited genes have been identified for the trait. To figure out the gene controlling the trait of exocarp color, we perform a comprehensive genome-wide association study, and we describe the candidate genes. One gene encoding the ABCG protein has been verified to be associated with the trait, using an integrative analysis of the metabolomic and transcriptomic data. This review covers a variety of omics resources, which provide a valuable resource for identifying gene-controlled traits of interest. The findings in this study help to elucidate the genetic components responsible for the trait of exocarp color in pear, and the implications of these findings for future pear breeding are evaluated.

## 1. Introduction

Pear (*Pyrus* L.) is a major fruit crop which has been cultivated in more than 80 countries. It is well liked for its being rich in juice, vitamins and minerals. The primary peel of the pear fruit is composed of four layers: the thick parietal cell layer, cork meristem, epidermal cells, and cuticle [1]. The occurrence of russet colors has been related to deficiencies and defects in the cuticle and epidermal layers [2,3,4]. Russeting is an important commercial surface defect which results in quality and price downgrading and plays a crucial role in attracting consumers and resisting undesirable environmental factors [5,6,7,8].

In recent decades, research referring to the transcriptome of russet fruit skin has been produced, and different gene expressions were found for the two varieties [9,10,11]. Compared with green peel, the expression of suberin deposition genes and stress-responsive genes was up-regulated in the russet group, and the expression of genes related to cuticle biosynthesis was down-regulated [12,13]. And the ATP-binding cassette (ABC) transporters involved in the transmembrane transport of cutin, suberin and lignin precursors have been shown to be involved in the regulation of exocarp pigmentation [14,15,16,17].

Testcross and QTL (quantitative trait locus) mapping studies have also been applied to the exocarp color trait in pear, while the corresponding genes were still uncertain, and the exact position of the gene controlling the trait was still unclear [18,19,20,21,22,23]. The availability of a wide range of genetic variants based on the development of whole-genome resequencing technology provides a tremendous opportunity to explore key genes for important traits using single-nucleotide polymorphisms (SNPs) for genome-wide association studies (GWAS). GWAS is an effective way to explore genome-level genetic architecture, and has been widely used to explore disease-associated genes in humans [24,25]. The method has also been used in plant population studies to explore candidate loci associated with complex traits, and to help identify genomic loci underlying key genes responsible for agronomic traits in pear [26,27,28,29,30].

In this study, we conducted GWAS for the exocarp color trait on a diverse group of 447 pear accessions. Genomic loci and the candidate genes responsible for the phenotypic traits collected for three successive years were determined using a GWAS analysis. We carried out the RNA-Seq project on russet and green peels of pears using Illumina sequencing technology. Metabolomics data were also used to analyze the content changes in the two varieties. By integrating the multiple omics, a gene from the ABC transporter G (ABCG) family was identified to function in pear exocarp color.

## 2. Materials and Methods

### 2.1. Plant Materials and Treatments

A total of 447 accessions, which are preserved in the Chinese National Germplasm Repository of Pear and Apple, Research Institute of Pomology, Chinese Academy of Agricultural Sciences (CAAS), were used as the research materials. Among the 447 accessions, 363 were collected from China, and 84 were collected from other countries. The measurement of the trait was based on the previously published *Description and Data Standard for Pear (Pyrus L.)* [31] and *Descriptors for Pear Germplasm Resources* (NY/T 2922-2016) [32]. The measurements were observed for three consecutive years.

Genomic DNA was extracted from the fresh leaves with the CTAB (hexadecyl trimethyl ammonium bromide) method [33]. For each accession, a sequencing library was constructed using at least 6 µg genomic DNA, according to the manufacturer’s instructions for the TruSeq nano DNA kit (Illumina, San Diego, CA, USA). The paired-end sequencing libraries were sequenced on NovaSeq6000 (150PE) from Berry Genomics Co., Ltd., Beijing, China.

### 2.2. SNP Detection and Annotation

The paired-end reads were firstly mapped to the reference genome of *P. betuleafolia*-Shanxi Duli (*Pbe*-SD) [34] using Burrows–Wheeler aligner software (BWA mem (version 0.7.12)). The HaplotypeCaller module in GATK (version 4.0.3.0) was used for local realignment [35]. To reduce the variant false discovery rate, the sites were subsequently filtered using the SelectVariants and VariantFiltration packages in Vcftools software (version 0.1.13) [36]. All identified SNPs that passed quality screening were further annotated using ANNOVAR, based on the reference genome [37].

### 2.3. Genome-Wide Association Study

The population structure was corrected using the kinship (K) matrix generated by the FaST-LMM program (version 0.4.1) [38]. GWAS analyses were implemented using a linear mixed-model algorithm from the single SNP module in the FaST-LMM program, which enables efficient genome-wide association studies on extremely large datasets. The cut-off used was *p* = 1 × 10^−6^; that is, the loci with significant thresholds higher than 6 were adopted for subsequent analysis.

### 2.4. Transcriptome Analysis

Green and russet samples were harvested separately at maturity, every 3 replicates, and immediately stored in liquid nitrogen. The total RNA of each sample was 2 µg, and was used as an input material for sample preparation. RNA purity and concentration were examined using NanoDrop 2000 (Thermo Fisher Scientific Inc., San Jose, CA, USA), and the integrity and quantity were measured using the Agilent 2100/4200 system. Library preparation and paired-end sequencing (2 × 150 bp) using high-quality RNA on Illumina’s NovaSeq platform were implemented, as recommended by the manufacturer. Raw reads were first processed through primary quality control and aligned against the *Pbe*-SD using HISAT2 (version 2.21) [39]. featureCounts (version 2.0.2) [40] was used to count the number of reads for each gene. A differential expression analysis of the ten genes selected in the GWAS study was performed using edgeR (version 3.3.3) [41]. Differential expression genes (DEGs) were defined as genes with adjusted *p*-values below 0.05 and log2 (fold change) values more than 1. Sequence alignment was implemented using DNAMAN (version 6.0.3) software. The Berry Genomics Corporation (Beijing, China) conducted the transcriptome analysis.

### 2.5. Expression Analysis Using qRT–PCR

RNA was isolated with an extraction kit (TIANGEN, Beijing, China); cDNA synthesis was performed using a cDNA synthesis kit (Takara, Dalian, China). The candidate gene sequences were obtained from the Genome Database for Rosaceae, “GDR; www.rosaceae.org (accessed on 25 April 2023)”. The primer used for gene expression analysis was as follows: Forward (5′ to 3′): TGACGACCTCTTCTTACTATC, Reverse (5′ to 3′): TGACGACCTCTTCTTACTATC. A quantitative real-time polymerase chain reaction (qRT-PCR) was carried out with a LightCycler System (Roche LightCycler96; Roche Diagnostics, Basel, Switzerland), following the manufacturer’s protocol. The expression levels of genes were calculated using the 2^−ΔΔCq^ method [42].

### 2.6. Metabolomics Analysis

The freeze-dried fruit samples were crushed and dissolved in aqueous methanol. After 6 vortex cycles (30 s/30 min), the samples were extracted overnight at 4 °C, then absorbed and filtered with a 0.22 μm filter (SCAA-104, ANPEL, Shanghai, China), and then a UPLC-MS/MS analysis was performed. Next, the sample extracts were analyzed using a UPLC-ESI-MS/MS system (UPLC, SHIMADZU Nexera X2, www.shimadzu.com.cn/ (accessed on 25 April 2023); MS, Applied Biosystems 4500 Q TRAP, www.appliedbiosystems.com.cn/ (accessed on 25 April 2023)). Significantly regulated metabolites between groups were identified as |Log2-ratio| ≥ 1 and VIP (variable importance in projection) ≥ 1, and then were annotated (http://www.kegg.jp/kegg/compound/ (accessed on 25 April 2023)) and mapped to the KEGG Pathway database (http://www.kegg.jp/kegg/pathway.html (accessed on 25 April 2023)). Metabolite set enrichment analyses (MSEA) were carried out to identify mapped pathways with significantly regulated metabolites. Correlation analyses between genes and metabolites were carried out with the R package in pheatmap (version 3.5.1). Metware Biotechnology Co., Ltd. (Wuhan, China) conducted the metabolomics detection and analysis.

## 3. Results

### 3.1. Genotyping of 447 Pear Accessions

**Figure 1:**
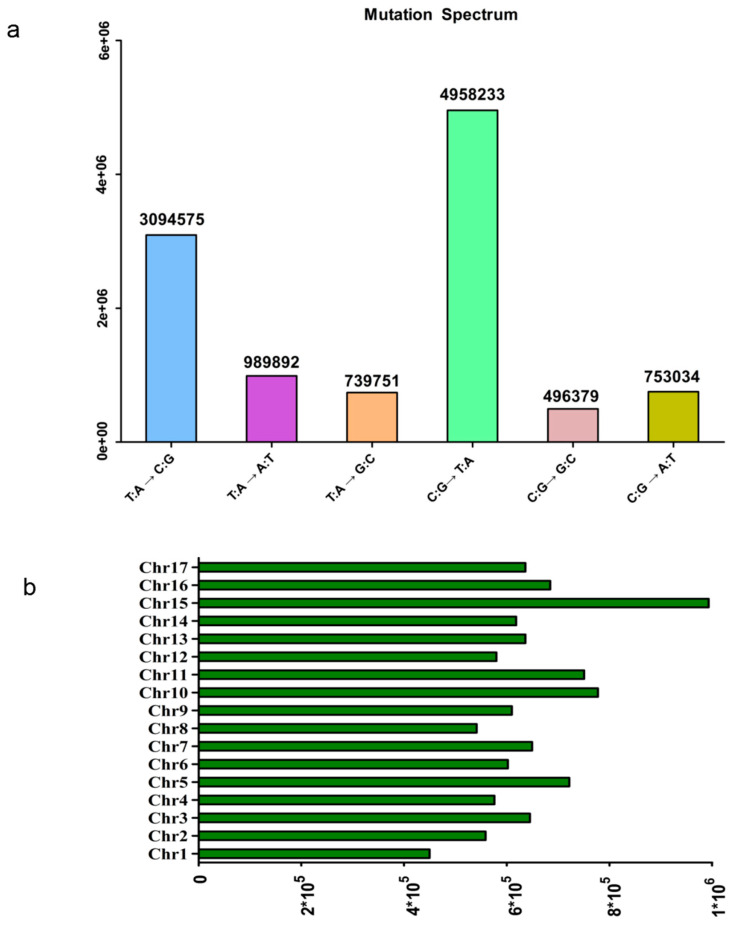
The distribution of the single-nucleotide polymorphisms (SNPs). (**a**) The distribution of different mutation types. (**b**) The number of SNPs across each chromosome of pear.

In our study, high-quality whole-genome resequencing was adopted for 447 pear accessions. A total of 24.71 billion paired-end read fragments and 3.7 terabase pairs (Tbp) of sequenced data were generated, resulting in 83.97% of reads mapped and a horizontal genome coverage of 84.12%. Eleven representative landraces were selected and mapped to two published pear genomes: the wild pear genome, *Pbe*-SD, and the Asian pear genome, ‘Dangshansuli’ [43], to select the optimal reference genome. Both the mapping rate and mean coverage of *Pbe*-SD were more ideal (Table 1), and *Pbe*-SD was finally chosen as the reference genome. After aligning the cleaned reads to the reference genome, we obtained an average sequencing depth of 10.76-fold, ranging from 7.46- to 21.88-fold. After SNP mapping and calling, a total of 11,031,864 high-quality SNPs were obtained for GWAS analysis. The sites with the highest mutation frequency were C:G~T:A (Figure 1a). And the distribution of SNPs across the genome was variable. Chromosomes 1 and 8 (Chr.1 and 8) had the most notable difference and had the fewest SNPs (449,759 and 541,431, respectively) compared with those of chr. 10 and 15 (777,524 and 993,236, respectively) (Figure 1b).

### 3.2. GWAS Analysis

**Figure 2:**
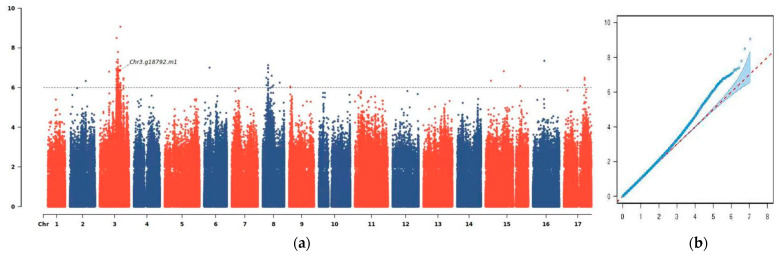
Genome-wide association study of the trait of exocarp color. (**a**) Manhattan plot of the trait of exocarp color. (**b**) Quantile–quantile plots of the observed *p*-values versus the expected values of associated *p*-values.

The significant associations between the SNP sites and the phenotype are shown in the global Manhattan plot (Figure 2a), from which the target sites can be quickly found, and the significance of the specific locations is clearly shown. Furthermore, the quantile–quantile plot shows that the observed *p*-values have significant deviations from the null distribution at the tail of the distribution (the distribution expected if there was no association) (Figure 2b). This indicates that the observed *p*-values, especially those at the tail of the distribution, are smaller than expected by chance. This probably suggests there is a significant correlation of natural selection between the phenotypes and genotypes studied in this research.

### 3.3. Identification of Putative Candidate Genes for Exocarp Color

The GWAS analysis revealed two obvious peaks on chromosomes 3 and 8 with genome-wide significance (*p* < 1 × 10^−6^). We subsequently searched the candidate genes in the surrounding space ±10 kb on either side of the significant SNPs. A total of 83 genes were found zooming the 40 significant SNPS, while 10 genes (*Chr6.g52876*; *Chr3.g19750*; *Chr3.g19751* et al.) surrounded by 16 significant SNPs were believed to be putative candidate genes related to exocarp color and were used in the subsequent analysis (Table 2). Among the 16 SNPs, some were found to be associated with one candidate gene (Table S1), such as Chr3:19122459, Chr3: 19129244, Chr3: 19131097, Chr3: 19125888, Chr3: 19127879, Chr3:19130039 and Chr3:19132108, which were associated with the gene *Chr3.g18987*. And Chr8: 5830002 and Chr8: 5827635 were associated with gene *Chr8.g54414*. Chr3: 19174112 and Chr3: 19174857 were associated with gene *Chr3.g18977*. And the function of the discovered susceptibility genes was as follows. *Chr6.g52876* encodes a member of the ethylene-responsive transcription factor involved in the path of biological and abiotic stress signal transduction in plants. And the relationship between ERF genes and russet peel has been studied in the previous study [44,45]. *Chr3.g19750* and *Chr3.g18792* are members of the ABCG family and play important roles in the formation of the protective layer and lipid exports [46]. *Chr3.g19751* is involved in the cell cycle and cell division, which are integrated with all of the processes of cell growth in multicellular organisms. The gene *Chr8.g54414*, encoding putative cytochrome P450 81e8, was attributed to drought and salinity stress responses [47]; the exocarp color trait is susceptible to these environmental factors. *Chr3.g18954* encodes hexokinase, which is widely known to participate in glycometabolism and is closely related to the energy utilization, biosynthesis and REDOX capacity of cells. *Chr3.g18987* encodes phytochromobilin, which regulates fruit chloroplast biogenesis and produces photo-assimilate products via fruit photosynthesis [48]. *Chr3.g19749* acts on the process of flavonoid biosynthesis and participates in the synthesis of various substances [49]. *Chr3.g18977* encodes scarecrow-like proteins which play an essential role in light signaling and substance transport [50,51]. *Chr3.g18343* encodes a protein containing the NAC domain that regulates a wide range of biological processes in plants [52].

### 3.4. Transcriptome Profiles for the Exocarp Color of Pear Fruit

**Figure 3:**
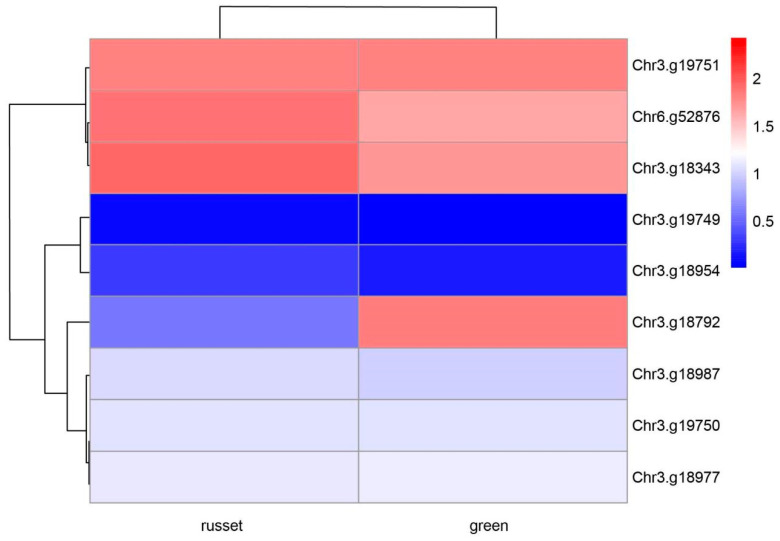
Hierarchical clustering analysis of genes picked using GWAS and transcription analysis.

To further investigate the effect of the 10 genes on the color of pear exocarp, a full-length transcriptome library was constructed using green and russet pear exocarp as materials. A total of 48 Gb clean sequencing reads were obtained for all the samples in the RNA-Seq analysis. In this study, the bio-repeats of all samples were R^2^ > 0.95, and the correlation coefficients between the samples were all above 0.90. According to the principle of *p* < 0.05 and log2 (Fold change) greater than 1, the expression levels of the ten genes between the green and russet samples were estimated. It was found that Chr3.g18792 was the unique DEG among the ten genes, while the other nine candidate genes were not (Figure 3; Table 2). Next, we used a parameter-free method to splice the CDS sequence of Chr3.g18792, and the results are shown in Figure S1. It was found that the different expression patterns observed in green and russet exocarp were caused by base mutations or upstream and downstream regulation.

### 3.5. qRT-PCR Analysis

**Figure 4:**
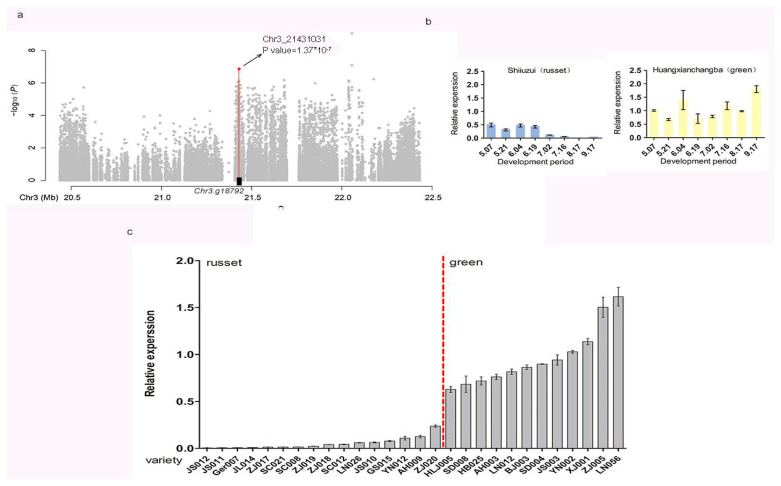
Local Manhattan plot for exocarp color and expression profiles of the candidate genes in pear. (**a**) Local Manhattan plot for GWAS study of exocarp color. The associated SNP located 8050 bp from Chr3.g18792 (chromosome. 3: 21,431,031 bp) is marked by a red dot. (**b**) Relative expression of the candidate gene Chr3.g18792 in russet and green exocarp along the whole development period at two-week intervals, detected using qRT-PCR. Russet and green varieties are indicated by blue and yellow, respectively. (**c**) Expression of the candidate gene Chr3.g18792 at maturity in russet and green varieties. Data are represented as average values with standard deviation of triplicates for qRT-PCR.

To verify the accuracy and reproducibility of the Illumina RNA-seq results, the Chr3. G18792 with altered transcript abundance between green and russet exocarp was selected for qRT-PCR analysis. The specific primer sequences of the gene were designed with the primer Premier 6 and tested to ensure the successful amplification of single discrete bands without primer dimers. The qRT-PCR results showed that the relative expression levels of this gene in green and russet exocarp were consistent with those of the RNA-seq results. It was shown that Chr3.g18792 had a higher expression in Huangxian Changba with green exocarp (*P. pyrifolia*; green) than Shiliuzui with russet exocarp (*P. pyrifolia*; russet) at all development stages (Figure 4b). Also, higher expression was found in 13 green exocarp cultivars in the mature stages (Figure 4c).

### 3.6. Comparison of Different Expression Metabolites between the Green and Russet Group

**Figure 5:**
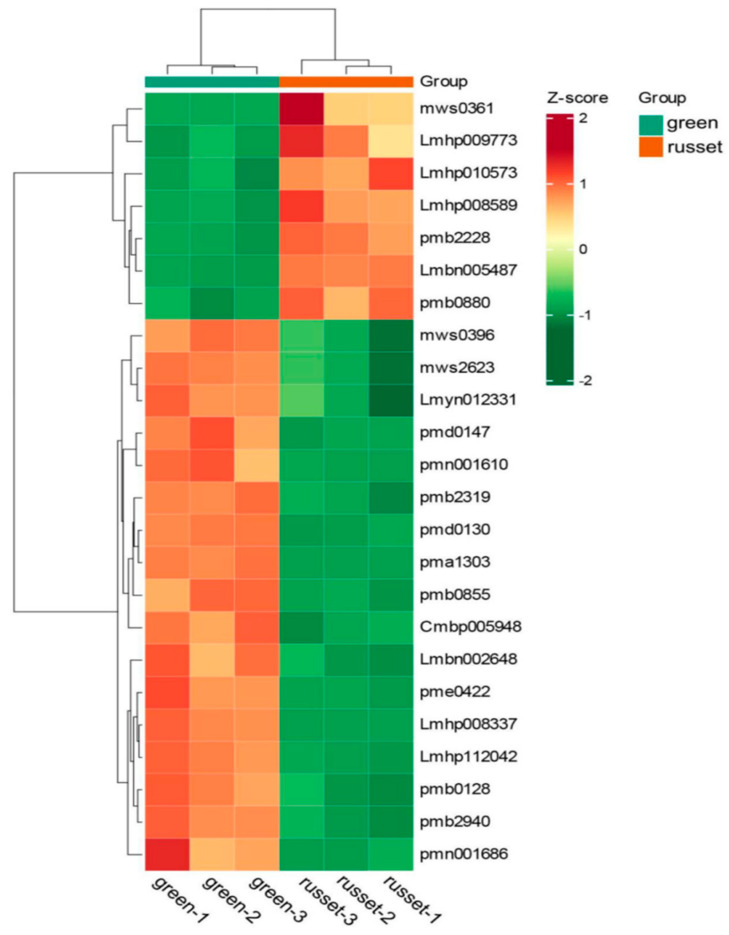
Cluster heat map of the 24 DEMs in the two varieties of exocarp color in pear.

The metabolites were screened with a cut-off of |Log2-ratio| ≥ 1 and VIP (variable importance in projection) ≥ 1 to identify the differentially expressed metabolites (DEMs) between green and russet pear exocarp. Compared with the green group, 228 DEMs were identified in the russet group, of which 92 were up-regulated and 136 were down-regulated (Table S2). And a total of 24 DEMs related to fruit exocarp color were selected and used for the subsequent study (Table S3). The expression patterns of the 24 DEMs in the russet and green groups, comparing three replications, are shown in Figure 5. There was a high degree of consistency between the three repetitions. And the distinctions of the 24 DEMs were exhibited between the two groups, among which 7 and 17 DEMs were up- and down-regulated in the russet varieties, respectively, while 17 and 7 were up- and down-regulated in the green varieties, respectively.

### 3.7. The Pathways Enriched by the 24 Differentially Expressed Metabolites Related to Exocarp Color

**Figure 6:**
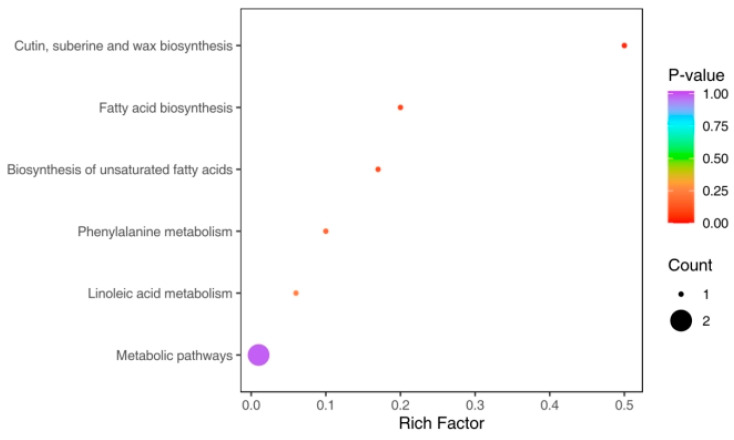
KEGG enrichment of DEMs between russet and green fruits.

The 24 differential metabolites selected were annotated using the Kyoto Encyclopedia of Genes and Genomes (KEGG) database. The KEGG enrichment analysis results of the selected DEMs are highly consistent with the previous analysis results. Consequently, the DEMs in the comparison group were assigned to the fatty acid elongation; cutin, suberine, and wax biosynthesis (ko00073); phenylalanine metabolic pathways (ko00360); fatty acid biosynthesis (ko00061); biosynthesis of unsaturated fatty acids (ko01040),]; linoleic acid metabolism (ko00591); and metabolic pathways (ko01100) (Figure 6, Table S4). *Chr3.g18792* encodes the ABCG15 protein, which is thought to affect the transport of wax and cutin and the formation of anther cuticles in rice [53]. And it has been reported that the ABCG15 gene in *Arabidopsis* has the closest homolog to the CER5 gene, which is involved in the biosynthesis of cuticular wax. The expression level of *Chr3.g18792* was found to be positively correlated with DEMs associated with the pathway of cutin, suberine and wax biosynthesis (ko00073) in our study (Figure S2). In summary, the results supported that *Chr3.g18792* may be involved in cutin formation, and is therefore linked to the occurrence of russet exocarp color in pear.

## 4. Discussion

Pear russeting occurs in most pear varieties in China and seriously affects the yield and quality of pear. Russeted fruits tend to lose market value. And it has been revealed that suberin, cutin, wax and lignin biosynthesis are related to russeting. Transcriptome studies on russeting in pear, apple and grape cultivars show that the formation of fruit russeting is regulated by a complex network, and the relevant genes are mainly related to the biosynthesis of cutin, suberin and wax [54]. It has been indicated that the decrease in cutin biosynthesis might be the cause of exocarp russeting. Consequently, decreased expression of cuticle biosynthetic genes has been observed in russeting cultivars.

The GWAS methodology is a powerful tool for analyzing simple traits under additive genetic scenarios, as well as dissecting more complex genetic architectures. The basics of GWAS are to assess the association between each genotype marker and phenotype that are scored in a population with a large number of individuals. It provides a useful tool for mining the underlying genetics of the trait, and provides valuable initial insights for subsequent validation [55,56,57].

Using GWAS analysis, an ABCG gene was found to be associated with the fruit russeting trait in our study. The ABC transporter family is the largest family of proteins ever discovered and is present in all organisms, from bacteria to humans [58]. In all, more than 400 members of the ABC protein family have been characterized. The ABC subfamily G plays a crucial role in the synthesis of extra-cellular barriers, and has been identified to be involved in a variety of metabolic processes throughout the plant life cycle. Recent research has shown that members of the ABCG subfamily are critical for lipid export. In *Arabidopsis*, ABCG11 is involved in cuticle formation. RNAi-mediated gene silencing or knockout of the AtABCG11 gene results in a significant decrease in cuticle lipid metabolism in Arabidopsis [59,60,61,62]. By analyzing transcripts of AtABCG11, the authors suggest that this transporter, expressed in the epidermis of the plant’s aerial organs, is localized in the plasma membrane of these epidermal cells [60]. The *Arabidopsis* ABCG13 transporter is necessary for flower cuticle secretion and petal cuticle formation [16]. ABCG1 is required for suberin formation of the tuber periderm in potatoes [63].

Integrated metabolomics and transcriptomics were conducted in this study, and the molecular mechanisms behind the difference in pear exocarp color were clarified. As was shown in Figure 3, the *Chr3.g18792* gene mapped by GWAS was found to be over-expressed in the green exocarp varieties. The metabolome data showed that the disturbance of cutin, suberine and wax biosynthesis may account for the major mechanism of pear russeting. A correlation analysis between the *Chr3.g18792* and DEMs related to exocarp color suggests that the gene may play an important role in the formation of cutin, suberin and wax biosynthesis in pear. In past studies, the characterization and expression of the ABC family (G group) have been researched with regard to pear russeting. Ten ABCG genes have been screened from the transcriptome of ‘Dangshansuli’ pear, and its russet mutant ‘Xiusu’, has been verified. The ABCG15 gene was found to have a higher expression in ‘Dangshansuli’ with green exocarp during the whole fruit development, which coincides with the results of our study. Some PbABCG genes exhibited unchanged or down-regulated expression over time, suggesting that these genes may operate in other signal transduction pathways in the complex regulatory network of pear russeting [64]. The expression patterns of ABC transporter genes at the transcript level and protein level were observed for the russet and green fruit skin of sand pear; 18 ABC transporter genes were differentially expressed at the transcript level, and only one gene was differentially expressed at the protein level between the two types of fruit skin. This indicates that different types of ABC transporters may be involved in the exocarp color of pear, and the functional differentiation and cooperation between different members need to be further studied.

## 5. Conclusions

GWAS acts as a powerful tool for linking the genotype–phenotype map and mining genes underlying complex genetic architectures. In this study, the gene *Chr3.g18792* located on chromosome 3 was identified using GWAS. To further explore the biochemical basis of color differences in pear exocarp, we compared the widely targeted metabolome and transcriptome data. The results verify the difference in suberin, cutin, and wax biosynthesis between russet and green exocarp. And the *Chr3.g18792* was proven to be correlated with the formation of suberin and cutin. Together, these conclusions support that the gene Chr3.g18792 identified in our study has a vital impact on the formation of russet exocarp in pear. The results help clarify the genetic basis for the exocarp color trait in pear and should provide a theoretical basis for the molecular breeding of pear. At the same time, our study collected the largest pear sequence dataset reported to date, and credibly serves as a valuable resource for the identification of genes controlling traits of interest in pear.

## Figures and Tables

**Table 1:** The mapping rate and mean coverage of the two selected reference genomes.

	*Pbe*-SD	Dangshansuli	Genebank (Dangshansuli)
Mapping rate	81.73–85%	70.78–73.58%	70.78–73.58%
Coverage	81.6–88.71%	70.29–74.34%	70.29–74.34%

**Table 2:** Basic information about genes from the GWAS and transcription analysis. The russet and green varieties were used as case and control groups, respectively. The significance of the gene is measured by the value of log (fold change), *p*-value and q value.

Gene ID	Description	Case Vsctrl. Log (Fold Change)	Case Vsctrl. *P* Value	Case Vsctrl. Q	Case Vsctrl. Significant
*Chr6.g52876*	ethylene-responsive transcription factor 2-like	0.43	0.32	0.73	FALSE
*Chr3.g19750*	abc transporter g family member 3-like	–0.08	0.72	0.94	FALSE
*Chr3.g19751*	cell division cycle protein 48 homolog	–0.07	0.73	0.94	FALSE
*Chr8.g54414*	cytochrome p450 81e8-like	–	–	–	–
*Chr3.g18954*	hexokinase-3-like	1.03	0.14	0.49	FALSE
*Chr3.g18987*	phytochromobilin:ferredoxinchloroplastic	0.08	0.78	0.96	FALSE
*Chr3.g19749*	udp-glycosyltransferase 86a1-like	2.27	0.01	0.10	FALSE
*Chr3.g18977*	scarecrow-like protein 3	–0.18	0.47	0.85	FALSE
*Chr3.g18792*	abc transporter g family member 15-like	–4.73	1.24 × 10^−45^	1.90 × 10^−42^	DOWN
*Chr3.g18343*	nac domain-containing protein 72-like	0.50	0.17	0.56	FALSE

## Data Availability

All transcriptome datasets for this study have been uploaded to the NCBI SRA database (PRJNA1004416).

## Supplementary Materials

The supplementary materials can be downloaded at: https://www.mdpi.com/article/10.3390/genes14091827/s1, Figure S1: Sequence alignment of *Chr3.g18792*; Figure S2: Correlations between *Chr3.g18792* and selected-DEMs; red line and blue lines correspond to positive and negative correlations, respectively; Table S1: Significant SNPs associated with putative candidate genes associated with russet; Table S2: Identification of DEMs in the green and russet exocarp; Table S3: Basic information about selected DEMs related to russet; Table S4: 24 DEMs mapped to KEGG metabolic pathways. Table S5: Information about the genotypes used for the transcriptome profiles and the qRT-PCR analysis.
