# Supplementary figures and images for: Identification and Characterization of ABCG15—A Gene Required for Exocarp Color Differentiation in Pear
Source: Genes (Basel). 2023 Sep 21;14(9):1827. doi: 10.3390/genes14091827 (PMC10530978; doi:10.3390/genes14091827)

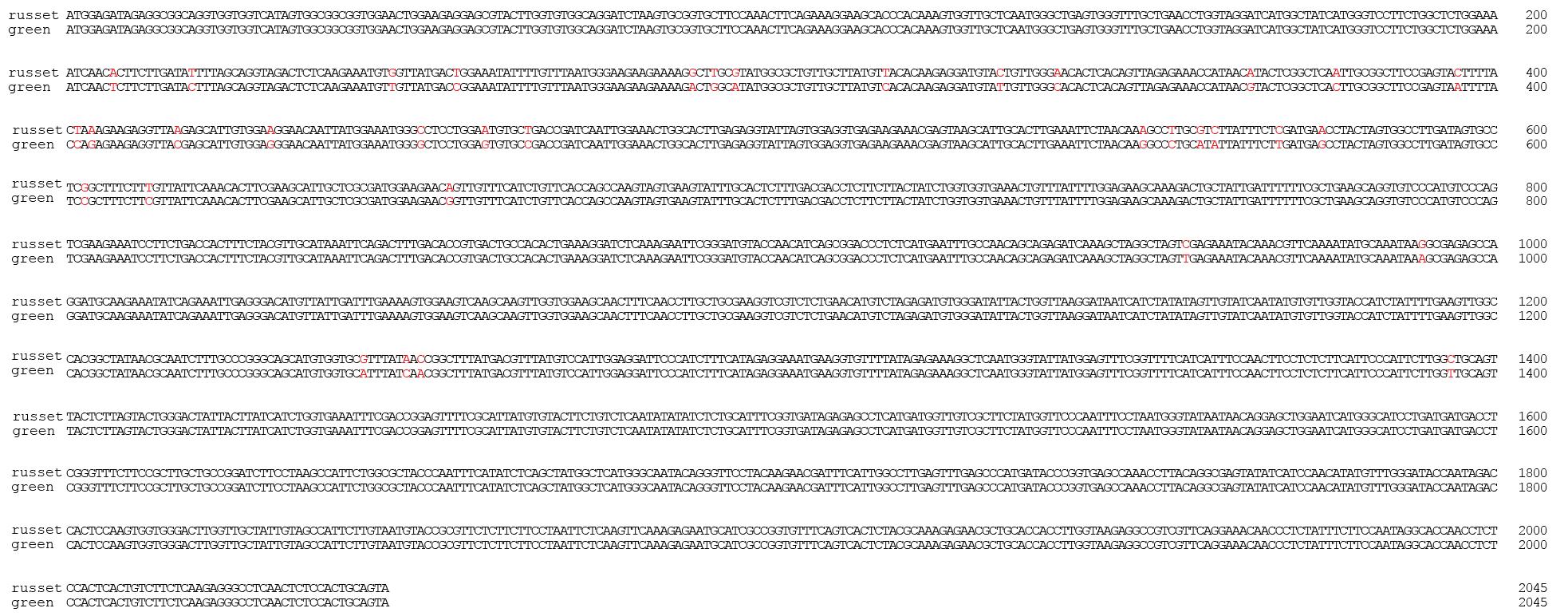

Supplement: Supplementary file 1 [file genes-14-01827-s001.zip › Figure S1.jpg]

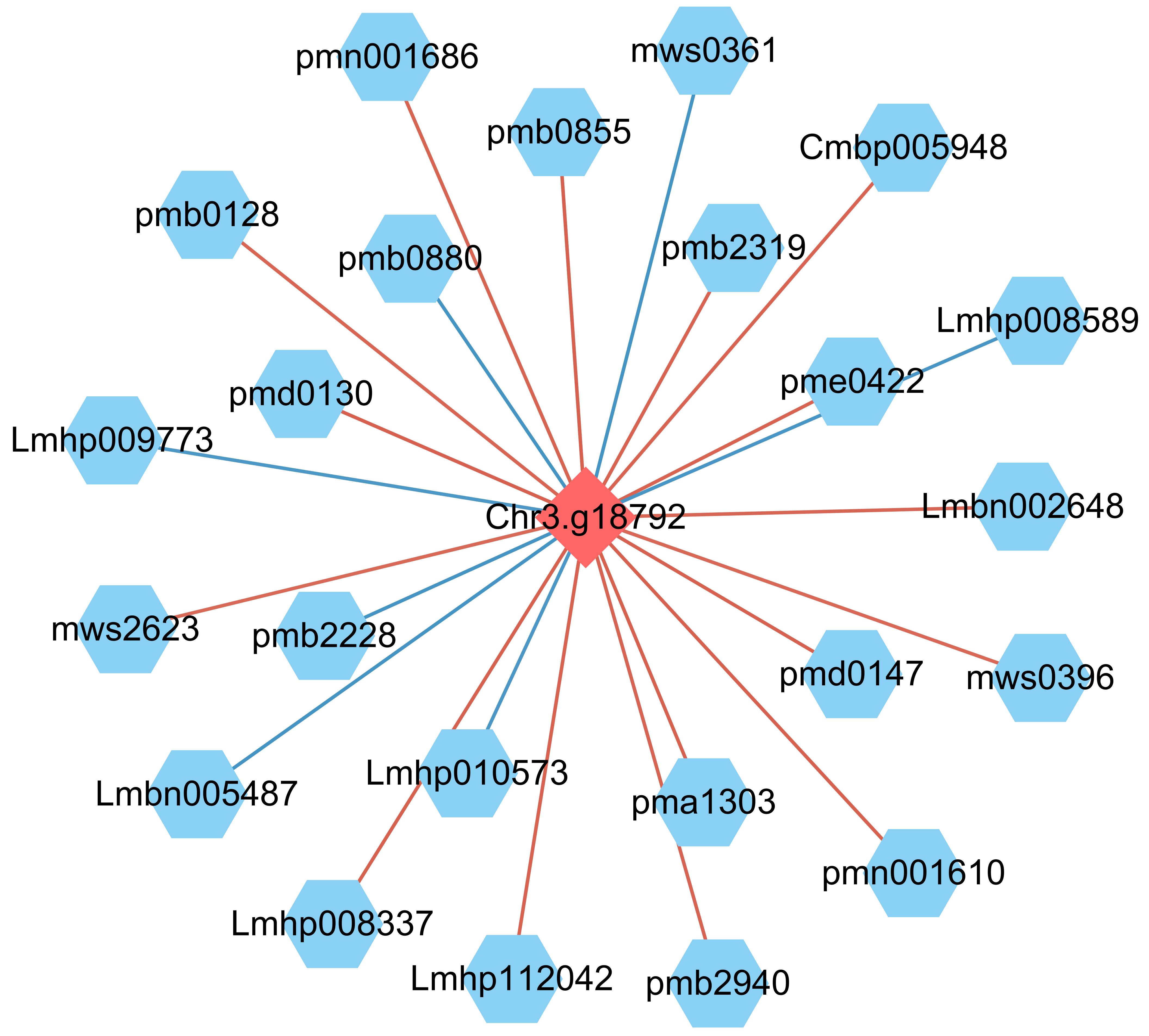

Supplement: Supplementary file 1 [file genes-14-01827-s001.zip › Figure S2.jpg]
